# Grape seed proanthocyanidin extract alleviates ouabain-induced vascular remodeling through regulation of endothelial function

**DOI:** 10.3892/mmr.2012.1026

**Published:** 2012-08-08

**Authors:** XIANGJU LIU, JIE QIU, SHAOHUA ZHAO, BEIAN YOU, XIANG JI, YAN WANG, XIAOPEI CUI, QIAN WANG, HAIQING GAO

**Affiliations:** 1 Department of Geriatrics, Shandong University Qilu Hospital, Jinan, Shandong 250012; 2 Shandong Provincial Key Laboratory of Cardiovascular Proteomics, Jinan, Shandong 250012, P.R. China

**Keywords:** grape seed proanthocyanidin extract, ouabain, rats, hypertension, vascular remodeling

## Abstract

Recent studies indicate that chronic ouabain treatment leads to hypertension and hypertensive vascular remodeling. Grape seed proanthocyanidin extract (GSPE) has been reported to be effective in treating arteriosclerosis, while little is known about its effect on systolic blood pressure and vascular remodeling. In this study, the effects of GSPE on systolic blood pressure and vascular remodeling were analyzed by treating ouabain-induced hypertensive rats with GSPE (250 mg/kg·d). The expression of nitric oxide (NO) and endothelin-1 (ET-1) in thoracic aorta was examined by ELISA; the mRNA and protein levels of TGF-β1 were detected using real-time PCR and western blotting, respectively. The results showed that the systolic blood pressure was significantly decreased following treatment with GSPE, with blocked vascular remodeling. The ET-1 content was reduced while NO production was increased in the GSPE group, which showed improved vascular endothelial function. Moreover, GSPE also reduced TGF-β1 expression in the thoracic aorta, which is a determinant in vascular remodeling. In conclusion, GSPE antagonized ouabain-induced hypertension and vascular remodeling and is recommended as a potential anti-hypertensive agent for patients with hypertensive vascular diseases.

## Introduction

Hypertension is a major health problem that leads to a range of diseases. Hypertension is capable of promoting vascular remodeling, which could be the main reason for increased peripheral vascular resistance and high blood pressure levels. Hypertensive vascular remodeling is associated with structural, functional and biochemical adjustments of endothelial cells, and it involves the degradation and reorganization of the extracellular matrix (ECM) scaffold, as well as hypertrophy and hyperplasia of the vascular smooth muscle cells (VSMCs), all of which contribute to a thickened vessel wall and augmented vascular stiffness.

Vascular cells (endothelial cells, smooth muscle cells and fibroblasts) are critical in vascular remodeling, and many studies suggested that the endothelium senses the hemodynamic changes and initiates the reorganization of the preexisting cellular and extracellular components. This remodeling involves cellular proliferation, apoptosis, migration, cell organization and matrix-integrin interactions throughout the layered structure of the vessel (1). Langille and O’Donnell demonstrated that the endothelium, or a substance produced by the endothelium, was essential for remodeling toward a smaller lumen after a long-term flow reduction (2).

The latest *in vitro* and *in vivo* studies have demonstrated that the transforming growth factor-β 1 (TGF-β1) isoform showed fundamental significance during vascular development, atherogenesis, neointima proliferation and vessel remodeling. The underlying mechanism may be related with its effects on regulating ECM synthesis, cell cycle progression, apoptosis, differentiation and migration (3–5). It has also been proven that gene expression of TGF-β1 may be associated with EC remodeling development (6).

Digitalis, which has been used in clinical practice for over 100 years, has a positive inotropic effect in myocardial cells. Ouabain, a digitalis compound, works as an endogenous regulator of blood pressure and Na+, K+-ATPase activity (7). Recent observations indicate that chronic ouabain treatment gives rise to hypertension (8) and hypertensive vascular remodeling (9).

The grape seed proanthocyanidin extract (GSPE) has been reported to be effective in treating arteriosclerosis (10), while little is known about its effects on systolic blood pressure and vascular remodeling.

In this study, the effects of GSPE on the blood pressure and vascular remodeling were examined by treating ouabain-induced hypertensive rats with GSPE (250 mg/kg·d), in tandem with the measurement of the systolic blood pressure and vascular remodeling parameters. The expression of nitric oxide (NO) and endothelin-1 (ET-1) in the thoracic aorta were examined by ELISA, and the mRNA and protein levels of TGF-β1 were respectively detected by real-time PCR and western blotting.

## Materials and methods

### Animals

A total of 30 male Sprague-Dawley (SD) rats (5–6 weeks old, weighing 180–220 g, supplied by the Experimental Animal Center of Shandong University, China) were housed in a 12:12-h light-dark cycle at 24°C, and had free access to tap water and standard rat chow *ad libitum* for 7 days to allow for acclimatization prior to entering the study. All protocols were approved by the Institutional Animal Care and Use Committee of the Qilu Hospital, Shandong University.

### Treatment

The 30 rats were randomized into three groups, with 10 rats in each group, treated with nitric sodium (NS), ouabain, GSPE and ouabain, respectively, and thus named the NS group, the O group and the GO group. Rats in the O group were administered ouabain (Sigma Chemical Co., St. Louis, MO, USA) at a dose of 27.8 *μ*g·kg^−1^·d^−1^ by intraperitoneal (ip) injection in the early morning of each day for 5 consecutive weeks. Rats in the GO group were administered oral GSPE at a dosage of 250 mg/kg·d, as well as ouabain at the same dose as the O group. Rats in the NS group were administered 0.9% saline (1 ml·kg-1·d-1) ip and 1 ml 0.9% NS orally for the same duration.

### Systolic blood pressure (SBP) measurement

The SBP of all animals was measured using an indirect tail-cuff plethysmographic (TCP) method with a rat tail BP monitor (RBP-I, Clinical Medicine Institute, Beijing Sino-Japan Friendship Hospital, China). The rats were kept calm and conscious until pulsatory signals from the arteria caudilis were displayed steadily. At least 10 determinations were made on each rat and the mean of 6 readings within a 5–10 mmHg range was taken as the SBP of the rat (11). The systolic and diastolic blood pressures were measured for 30 min with a pressure transducer (model 1050BP, UFI, Inc., Morro Bay, CA, USA) and then recorded using an interface and software for computer data acquisition (model MP100A, BIOPAC System, Inc. Santa Barbara, CA, USA). The SBP was measured once a week until the end of the experiment (six times in total).

### Tissue collection

Following five weeks of treatment, the rats were sacrificed by decapitation. Thoracic aortas were rapidly excised and dissected. For morphological and immunohistochemical examination, aorta were fixed in buffered 10% neutral formalin. For electron microscopy, thoracic aorta were trimmed to 1×1×1 mm and fixed in 2.5% glutaraldehyde solution. For analysis of TGF-β1 expression, the aorta were instantly frozen in liquid nitrogen and maintained at −80°C prior to use.

### Hematoxylin and eosin (HE) staining

Specimens of thoracic aorta were fixed into buffered 10% neutral formalin for 48 h, dehydrated in graded ethanol solutions, and then embedded in paraffin. The paraffin-embedded specimens were sectioned at 5 *μ*m. The morphological changes were examined by light microscopy following HE staining.

### Ultrastructural examination

Thoracic aorta were trimmed into tissue blocks of 1 mm^3^ on ice and immediately put into the 2.5% glutaraldehyde fixation solution at 4°C for 2 h followed by postfixation in 1% osmium tetroxide (in 0.1 M phosphate buffer) for 2 h at 4°C. The samples were then dehydrated in a graded ethanol series with acetone, permeated and embedded in epoxide resin. Semi-thin sections of approximately 75 nm were prepared, stained with uranyl acetate and lead citrate, and then observed with an H-800 transmission electron microscope (TEM; Hitachi Electronic Instruments, Tokyo, Japan).

### ELISA

Animals were treated as above, and thoracic aortas were excised and stored at −70°C until assay. The amount of NO and ET-1 was measured using a colorimetric method (optical density) after detection of the protein with a kit purchased from Shanghai Jianglaibio Ltd. Co. (Shanghai, China) and Enzo Life Sciences, Inc. (NY, USA), respectively.

### Total RNA isolation and cDNA synthesis of TGF-β1

Total RNA isolation from tissues was performed with TRIzol reagent (Omega Bio-Tek, Norcross, GA, USA) according to the manufacturer’s instructions. All total RNA samples were subjected to DNase I treatment (DNase I; Fermentas, Burlington, ON, Canada) and stored in RNase-free double distilled water at −80°C. RNA quantity and purity were determined by spectrophotometry. The integrity of RNA molecules was monitored by 1% agarose gel electrophoresis, and specimens with well-pronounced rRNA bands were selected for reactions. The first strand of cDNA was synthesized as follows (First Strand cDNA Synthesis kit, Fermentas): a mixture of 1 *μ*g of total RNA, 1 *μ*l oligo (dT)18 primer and DEPC-treated water to 12 *μ*l was heated for 5 min at 65°C and chilled on ice; then 4 *μ*l of 5× reaction buffer, 1 *μ*l of RNase inhibitor (20 U/*μ*l), 2 *μ*l of dNTP mix (10mM) and 1 *μ*l of reverse transcriptase (200 U/*μ*l) were added and incubated for 60 min at 42°C, followed by 5 min at 70°C to inactivate the reverse transcriptase, and the mixture was then stored at −20°C for mRNA expression analysis.

### Real-time quantitative PCR analysis of TGF-β1

Real-time quantitative PCR (qPCR) was performed by RealMaster Green (Tiangen, Beijing, China). The total reaction volume was 20 *μ*l (1.5 *μ*l of cDNA template, 8 *μ*l 2.5× real master mix, 1 *μ*l 20× SYBR solution, 9 *μ*l double distilled water and 0.25 *μ*l 5 mM each of the forward and reverse primers). The real-time quantitative PCR program was 95°C for 1 min, followed by 35 cycles of 95°C for 5 sec, 58°C for 15 sec and 68°C for 20 sec. Melting curve analysis and 2% agarose gel electrophoresis were used to confirm the specificity of each product, the efficiency of PCR was determined by analysis of two-fold or five-fold serial dilutions of cDNA and designed to detect all the signals in the spanning region. The efficiencies were close to 100%, allowing the use of the 2^−ΔΔCT^ method for calculation of relative gene expression. All qPCR was conducted with negative controls. The mRNA expression levels of TGF-β1 in the thoracic aorta were examined by qPCR. Each sample was performed in triplicate and the data were normalized to β-actin expression. The primer sequences are listed in Table I.

### Western blot analysis of TGF-β1 protein expression

Total protein was extracted from the frozen thoracic aorta tissues using RIPA lysis buffer (1% Triton X-100, 1% deoxycholate, 0.1% SDS) and 1 mM PVMF. Following ultrasonication for 5 min, extracts were centrifuged at 12,000 × g for 15 min at 4°C, and the supernatants containing protein were retained. The protein concentrations in the samples were measured with the BCA method (Beyotime^®^ Institute of Biochemistry, China). In total, 50 *μ*g of protein samples were resolved by electrophoresis on a 12% SDS-polyacrylamide gel (Bio-Rad, Hercules, CA, USA). Proteins were transferred onto a polyvinylidene difluoride (PVDF) membrane. After blocking with 5% skimmed milk/TBST for 1 h, the membranes were incubated overnight with primary antibodies against TGF-β1 (mouse monoclonal, 1:250, Abcam^®^, Hong Kong), and then stripped and incubated with the respective peroxidase-conjugated AffiniPure goat anti-rabbit/mouse IgG (1:10000, ZSGB-Bio). The bands were visualized using the enhanced chemiluminescence system (ECL) and analyzed densitometrically using Image J software. In the meantime, PVDF membranes were probed with β-actin as an internal control to ensure equal loading.

### Statistical analysis

All data analyses were performed using SPSS^®^ version 11.5 (SPSS^®^ Inc., Chicago, IL, USA) for Windows^®^. The data were shown as the means ± SD. An independent sample t-test was used to compare continuous data between the two groups. P<0.05 was considered to indicate a statistically significant difference.

## Results

### Blood pressure

**Figure 1:**
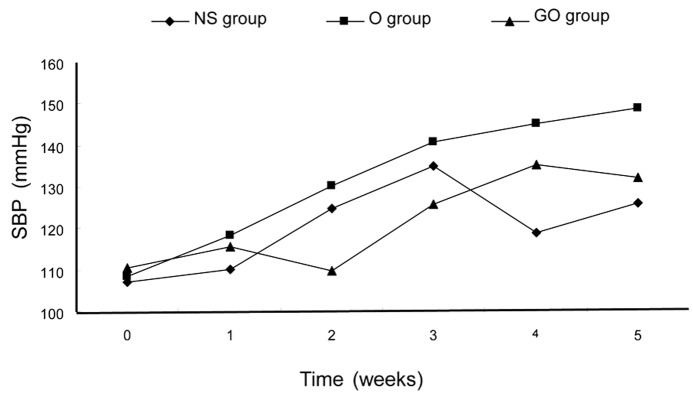
Changes in SBP in the three experimental groups over a 5-week period. SBP, systolic blood pressure; NS group, nitric sodium group; O group, ouabain group; GO group; grape seed proanthocyanidin extract and ouabain group.

Over a 5-week treatment period, the mean SBPs of the three groups were 108.1, 150.3 and 111.8 mmHg, respectively. There was no significant difference between the GO group and the control group (P>0.05; Fig. 1). However, in the O group, the systolic blood pressure was much higher than that of the GO and NS groups (P<0.05). At the end of the treatment, no significant difference in body weight among the three groups was observed (329±14, 335±9 and 317±10 g, P>0.05). Fig. 1 shows the changes in SBP in the three experimental groups over a 5-week period. SBP was assessed using the TCP measurements in each group. The blood pressure was the same in each group at the baseline (P>0.05), However, after 5 weeks, GSPE-treated animals (GO group) showed significantly decreased SBP compared with those in the O group (P<0.01).

### HE staining of aorta

**Figure 2:**
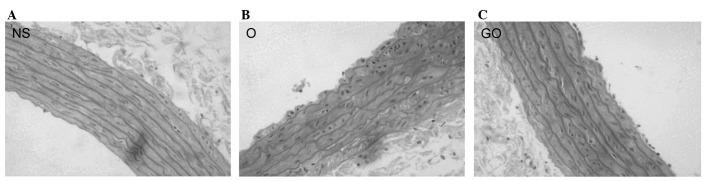
Histological sections of thoracic aorta stained with hematoxylin/eosin in the three groups (magnification, ×200). (A) NS group, nitric sodium group; (B) O group, ouabain group; (C) GO group; grape seed proanthocyanidin extract and ouabain group.

Fig. 2 shows histological sections of the thoracic aorta stained with HE. The arrangement of the elastic fibers of aortas from rats in the NS group was normal and there was no hyperplasia of collagen in the vessel wall (Fig. 2A). The aortic wall in the O group rats thickened, with hyperplastic collagen fibers in the media and with decreased, disordered and even ruptured elastic fibers (Fig. 2B). Aortic elastic fibers in the GO group were fairly ordered. Collagen fibers were almost normal compared to that in the O group (Fig. 2C).

### Ultrastructural changes of the thoracic aorta

**Figure 3:**
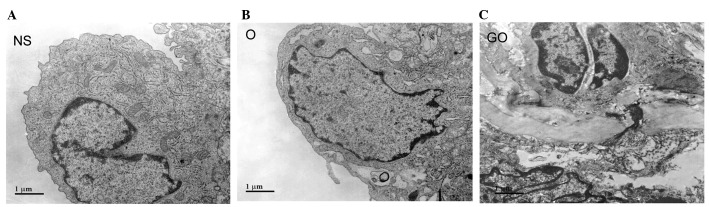
Transmission electron photomicrographs of thoracic aorta in three groups. (A) NS group, nitric sodium group; (B) O group, ouabain group; (C) GO group; grape seed proanthocyanidin extract and ouabain group.

Fig. 3 shows transmission electron photomicrographs of the thoracic aorta in the three groups. The NS group showed a normal tight junction and gap junction structure between the endothelial cells. The majority of heterochromatin was distributed in the circumference of the nucleus. In the O group, the morphology of endothelial cells in the thoracic aorta changed, with vacuolated cytoplasm and enlarged endoplasmic reticulum, and with no or decreased myofilaments. Nuclear chromatin was dense and observed in lumps of different sizes, which were mainly located in the nuclear membrane. In the GO group, elastin fibers among the endothelial cells increased, with irregular arrangement, and were partly disrupted. Nucleolemma introcession was observed.

### Expression of NO and ET-1

**Figure 4:**
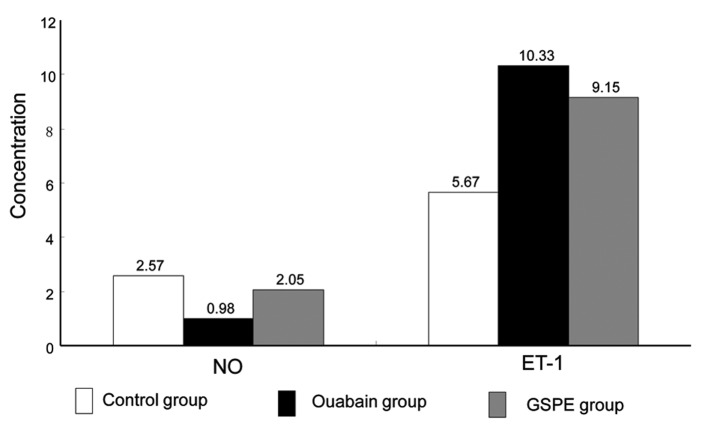
The concentration of NO and ET-1 in the thoracic aorta from the three groups. NO, nitric oxide; ET-1, endothelin-1; GSPE, grape seed proanthocyanidin extract.

Fig. 4 shows the concentration of NO and ET-1 in the thoracic aorta from the three groups. Compared with the NS group, the concentration of NO in the thoracic aorta in the O group decreased significantly (0.98 vs. 2.57 pg/mgprot, P<0.01); while GSPE treatment increased NO production when comparing the GO group with the O group (2.57 vs. 0.98 pg/mgprot, P<0.01). However, the ET-1 expression increased greatly in the O group in comparison to that in the NS group (10.33 vs. 5.67 pg/ml, P<0.01), which was capable of being reversed by GSPE treatment (10.33 vs. 9.15 pg/ml, P<0.01).

### Protein expression of TGF-β1

**Figure 5:**
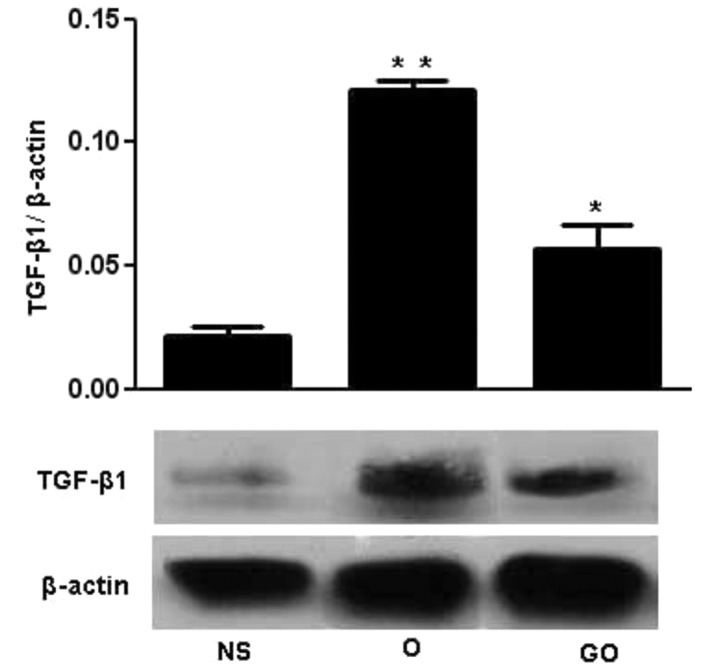
Western blot analysis of TGF-β1 expression in the three groups. NS group, nitric sodium group; O group, ouabain group; GO group; grape seed proanthocyanidin extract and ouabain group. ^*^P<0.05; ^**^P<0.01.

Fig. 5 shows western blot analyses of TGF-β1 expression in the three groups. Lysates of the aorta cells treated with or without GSPE were analyzed by western blotting using TGF-β1 antibody with β-actin as an internal control. TGF-β1 expression in thoracic aortas in the O group was significantly increased compared to that in the NS group, and GSPE could decrease the expression of TGF-β1 compared with that in the O group.

### Real-time quantitative PCR analysis of TGF-β1

**Figure 6:**
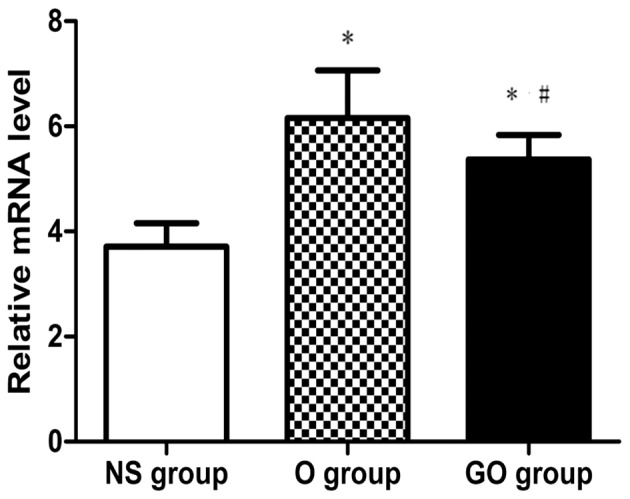
mRNA expression of TGF-β1 in three groups. ^*^P<0.05 compared with NS group. ^#^P<0.05 compared with O group. TGF-β1, transforming growth factor-β 1; NS group, nitric sodium group; O group, ouabain group; GO group; grape seed proanthocyanidin extract and ouabain group.

Fig. 6 shows the mRNA expression of TGF-β1 in the three groups. Total RNA was isolated from the thoracic aorta in NS, O and GO group rats and subjected to RT-PCR. 18S rRNA gene expression was used as an internal control. The results revealed that the mRNA expression of TGF-β1 in the O group was significantly increased compared to that in the NS group, and GSPE was capable of decreasing the mRNA level of TGF-β1 significantly compared with that in the O group.

## Discussion

Hypertension is a major factor promoting vascular remodeling, which leads to vascular stiffness. Vascular remodeling involves degradation and reorganization of the ECM scaffold, as well as hypertrophy and/or hyperplasia of the vascular smooth muscle cells (VSMCs). It was reported that VSMCs and cardiac hypertrophy were found before high blood pressure in the spontaneously hypertensive rat (SHR) without correlation with blood pressure levels (12).

Digitalis has a positive inotropic effect in myocardial cells, and it has been used in clinical practice for over 100 years. Ouabain, as a digitalis compound, is an endogenous regulator of blood pressure and Na+, K+-ATPase activity (7). Recent studies suggested that chronic ouabain treatment produced hypertension (8) and hypertensive vascular remodeling (9).

Elevated levels of endogenous ouabain or a closely related isomer are involved in rat and human hypertension and in associated cardiovascular complications. Several findings indicated that endogenous ouabain, in addition to directly influencing blood pressure, may be involved in the development of cardiovascular complications (cardiac hypertrophy, heart failure and myocardial infarction) associated with hypertension. Endogenous ouabain may therefore play an important role *in vivo* as a prohypertrophic hormone and thus may affect cardiovascular function and structure, as it is responsible for cardiac remodeling which contributes to an increased risk of morbid events (8,13–15). Furthermore, exogenous ouabain induced hypertension when chronically administered to normotensive rats (16,17).

Our study showed that a five-week ouabain administration could induce hypertension effectively. Moreover, histological studies showed that ouabain significantly promoted neointimal hyperplasia and VSMC migration when compared with those of the control group.

GSPEs are a group of polyphenolic bioflavonoids exhibiting multiple pharmacological activity and therapeutic potential (4,18). GSPEs have been reported to protect against oxidant injury during ischemia/reperfusion in the rat heart (19–21). Although previous studies have implicated antioxidant effects of GSPE (19–21), none of them revealed the effect of GSPE on endothelial function and hypertension.

In 2009, a study demonstrated that GSPE was capable of lowering blood pressure in subjects with metabolic syndrome and they also found that the phenolic compounds in the extract are absorbed and that its antioxidant properties were capable of reducing the concentration of Ox-LDL in plasma (22).

Endothelium-derived relaxing factors such as NO and prostacyclin usually act in coordination with endothelium-derived constricting factors such as ET-1, thromboxane and serotonin to accommodate changes in the cardiac output and to keep the blood pressure relatively constant. The imbalance of these endothelium-derived factors may elevate vasomotor tone, promote VSMC proliferation and induce vascular remodeling.

NO and ET-1 are key regulators of vasodilatory actions. NO acts as a second messenger for the actions of a number of growth factors, peptides, coagulation factors and hormones, and is a powerful regulator of vascular function. Endothelium-derived NO is a powerful regulator of vascular function, and it appears that the abnormalities in the production or actions of NO lead to endothelial dysfunction and abnormal vascular remodeling (1). ET-1 is the dominant vasoconstrictive factor. Studies have noted that aortic ET-1 content is significantly increased in DOCA-salt hypertensive rats compared with that in age-matched control rats (23). It has been proposed that this hypertension is due to an imbalance between endogenous vasoconstrictors and the diminished vasodilating effect of NO. Several candidates for endogenous vasoconstrictors that may contribute to sustained hypertension induced by NO blockade have been reported (24–26).

To evaluate the effect of GSPE on endothelial function, ELISA was carried out to examine the concentration of NO and ET-1 in the thoracic aorta.

Through detecting the concentration of NO and ET-1 in the thoracic aorta, we proved that ouabain impaired the balance between NO and ET-1, two major vaso-active substances, which may contribute to damaged endothelial function. GSPE increased NO production and decreased ET-1 expression, resulting in improved endothelial function and better vasodilation.

Improved endothelial function is capable of inhibiting vascular remodeling. Therefore, we subsequently detected another molecule (transforming growth factor-β 1, TGF-β1) that is critical in mediating vascular remodeling. The latest *in vitro* and *in vivo* studies also demonstrated that TGF-β1 is of fundamental importance during vascular development, atherogenesis, neointima proliferation and vessel remodeling. The possible mechanism may be due to its regulation of ECM synthesis, cell cycle progression, apoptosis, differentiation and migration (3–5). A previous study showed that gene expression of TGF-β1 may be associated with its development (6). A large number of studies reveal that increased mRNA levels of TGF-β1 were observed in myocardial remodeling (27).

In the present study, we proved that ouabain could induce TGF-β1 expression at the mRNA and protein level, resulting in vascular remodeling. This result was consistent with the previous reports. GSPE could efficiently inhibit this harmful pathway and therefore block the vascular remodeling induced by ouabain.

In conclusion, our present study suggested that GSPE could decrease blood pressure efficiently and reverse vascular remodeling in ouabain-induced hypertensive rats. This may be attributed to the regulation of NO and ET-1 balance and the suppression of TGF-β1 expression by GSPE. Therefore, GSPE may be a potential anti-hypertensive agent for patients with hypertensive vascular diseases.

## Figures and Tables

**Table I:** Primer sequences for detection of PCNA mRNA transcripts.

Gene	Sense (5′-3′)	Anti-sense (5′-3′)	Length (bp)
β-actin	GAAGTGTGACGTTGACAT	ACATCTGCTGGAAGGTG	245
TGF-β1	AGAAGTCACCCGCGTGCTAAT	CACTGCTTCCCGAATGTCTGA	144

PCNA, proliferating cell nuclear antigen; TGF-β1, transforming growth factor-β 1.
